# Neurodegenerative and Glial Physiology in Diabetic Retinopathy: Dissociated Effects of Forskolin on Neuronal Survival and Glial Activation

**DOI:** 10.3390/biomedicines14051104

**Published:** 2026-05-13

**Authors:** Hesham Saad Ata, Nessren M. Abd el-Rady, Asmaa M. S. Gomaa, Ahmed F. Omar, Ahmed Abdou, Maha Ali, Shimaa E. Soliman, Nada M. Fathy, Marwa H. Bakr, Dalia A. Elgamal, Manal M. Sayed, Eman Radwan, Amel Ahmed

**Affiliations:** 1 Department of Pathology, College of Medicine, Qassim University, Qassim 51452, Saudi Arabia; mahaali@aun.edu.eg (M.A.); s.saad@qu.edu.sa (S.E.S.); 2 Department of Medical Physiology, Faculty of Medicine, Assiut University, Assiut 71515, Egypt; nessren@aun.edu.eg (N.M.A.e.-R.); asmaasayed@aun.edu.eg (A.M.S.G.); 3 Department of Medical Physiology, Sphinx University, New Assiut, Assiut 71684, Egypt; 4 Department of Physiology, College of Medicine, Jouf University, Sakaka 72388, Saudi Arabia; 5 Department of Ophthalmology, Faculty of Medicine, Assiut University, Assiut 71515, Egypt; ahmed.omar@aun.edu.eg (A.F.O.); abdou.ahmed@aun.edu.eg (A.A.); 6 University Hospitals Eye Institute and the Department of Ophthalmology and Visual Sciences, Case Western Reserve University School of Medicine, Cleveland, OH 44106, USA; 7 Department of Medical Biochemistry and Molecular Biology, Faculty of Medicine, Assiut University, Assiut 71515, Egypt; emanradwan@aun.edu.eg; 8 Faculty of Medicine, Assiut University, Assiut 71515, Egypt; naddafathy@gmail.com; 9 Department of Histology and Cell Biology, Faculty of Medicine, Assiut University, Assiut 71515, Egypt; m.elbadawy@aun.edu.eg (M.H.B.); manalgomea@yahoo.co.uk (M.M.S.); 10 Department of Basic Medical Science, Badr University in Assiut BUA, Assiut 51711, Egypt; dalia.elgamal@bua.edu.eg; 11 Department of Biochemistry, Sphinx University, Assiut 71684, Egypt; 12 Department of Pathology, University Hospitals Cleveland Medical Center, Case Western Reserve University School of Medicine, Cleveland, OH 44106, USA

**Keywords:** biomarkers, disease exacerbation, caspase 3, DR, FSK, GFAP, IBA1, miR-200b

## Abstract

**Background/Objectives**: Using a well-established model of streptozotocin-induced diabetic retinopathy (DR), this study sought to evaluate the neuroprotective effect of intravitreal Forskolin (FSK) on retinal ganglion cell survival and glial activation and explore the association of circulating miR-200b with metabolic and oxidative stress in DR. **Methods**: A total of 18 male Wistar rats were divided into a control group (n = 6) and a streptozotocin-induced diabetic group (n = 12), which were further divided into diabetic control and FSK-treated groups (n = 6 each). Total antioxidant capacity (TAC), total peroxide (TP), triglycerides (TGs), total cholesterol, and high-density lipoprotein cholesterol (HDL-C) were measured. qRT-PCR analysis for miRNA-200b and immunohistochemistry were performed. **Results**: Diabetic rats showed oxidative stress and hyperlipidemia associated with increased circulating miR-200b levels. The retina showed reduced neuron numbers (Caspase-3), altered glial and astrocyte staining (IBA1, GFAP), and changes in microglia/macrophage morphology and distribution. Intravitreal FSK improved retinal ganglion cell survival and reduced glial activation, while systemic lipid profile and oxidative stress markers remained largely unchanged. Circulating miR-200b levels showed a positive correlation with oxidative stress markers across groups. **Conclusions**: Intravitreal FSK was able to limit the disease exacerbation via improved neuronal survival through inhibition of apoptosis. FSK did not produce observable qualitative changes in GFAP expression or IBA1+ cell morphology under the conditions tested.

## 1. Introduction

Diabetic retinopathy (DR) is the most common ocular complication of diabetes mellitus [1] and one of the leading causes of secondary blindness worldwide [2]. Studies have shown that the primary pathology in DR is related to the dysfunction and death of retinal ganglion cells (RGCs) [3]. Apoptosis is an important mode of cell death and a key mechanism in the development and progression of DR. Caspase 3, an important protein regulating apoptosis [4], is thought to be an important pathway for the cascade of apoptosis proteases and is involved in the onset of DR and its severity [5]. Neuroinflammation also plays a remarkable role in the pathogenesis of DR, with the activated glia contributing to the inflammatory response expansion [6]. Since the retinal neuron cannot regenerate after injury, the RGC loss is irreversible and results in permanent visual impairment [7]. The current therapeutics for DR treat the associated complications as retinal neovascularization, but do not promote functional recovery [1]. Although longer diabetes duration, poor glycemic control, high blood pressure, and hyperlipidemia are considered major risk factors for DR [8,9,10], epidemiological data support the hypothesis of differential genetic susceptibility to this chronic complication [9,11].

MicroRNAs (miRNAs) are small non-coding RNAs capable of regulating gene expression, resulting in transcript degradation or translational suppression. In the whole genome, about 30% of the genes are subjected to miRNA regulation [12]. Given their high stability and omnipresence in body fluids, several studies have identified the potential of circulating miRNAs as biomarkers of diagnosis, prognosis, and management of diabetes and its vascular complications [13,14,15]. MiR-200b is a recent miRNA found to be altered in high glucose-exposed retinal cells and diabetic murine models, thereby suggesting its contribution to DR development [16]. However, to the best of our knowledge, no study to date has assessed the circulating levels of this miRNA in diabetic rats as a possible diagnostic marker of DR.

Forskolin (FSK) is a natural diterpenoid extracted from Coleus forskohlii [17]. FSK is an adenylate cyclase activator that has been shown to promote neuronal survival by stimulating neurotrophic activity [18]. In Alzheimer’s disease models, FSK can attenuate microglial and astrocyte activation in the cortex and provide neuroprotection [17]. Moreover, FSK inhibits macrophage activation with a subsequent reduction in superoxide levels, suggesting its antioxidant effects [19]. Interestingly, FSK can inhibit the retinal glucose uptake by glucose transporter 1 (GLUT1), which is the only transporter that allows glucose to pass through the blood–retinal barrier [20]. In this study, using a well-established model of streptozotocin (STZ)-induced DR, we aimed to investigate retinal changes, RGC counts, glial cell activity, plasma oxidative stress markers, lipid profile, and circulating miR-200b levels as a potential diagnostic marker of DR. We also evaluated the neuroprotective effect of FSK and explored whether its retinal effects were associated with systemic oxidative stress and miR-200b levels.

## 2. Materials and Methods

### 2.1. Animals

A total of 18 young adult male Wistar albino rats, about 8–10 weeks old, weighing 200–300 g, were used. The rats were divided into two main groups: Group I (control group, n = 6), which received an intraperitoneal (IP) injection of 0.5 mL of sterile saline 0.9%; Group II (diabetic group, n = 12), which received an IP injection of STZ (100 mg/kg) to induce diabetes mellitus. Group II was further divided into: Group IIa (diabetic control group (DM); n = 6), in which the rats were sacrificed eight weeks after diabetes induction to obtain blood samples, and Group IIb (diabetic Forskolin group (DM + FSK); n = 6), which received an intravitreal injection of FSK (6 nmol/eye) in the right eyes 8 weeks after diabetes induction, while the contralateral left eyes received vehicle injection as controls.

Rats with fasting blood glucose <200 mg/dL after STZ injection, or those showing lens injuries, cataracts, or vitreous hemorrhage after intravitreal injection, were excluded.

All rats were housed in the Central Animal House, Faculty of Medicine, Assiut University, under a 12 h light/dark cycle with ad libitum access to food and water. Experimental protocols were approved by the Institutional Animal Care and Use Committee at Assiut University and conducted in accordance with the Guide for the Care and Use of Laboratory Animals (Approval number:17300174).

### 2.2. Diabetes Induction

The rats were fasted 12 h before STZ (Sigma Chemical, St. Louis, MO, USA) injection; then, each animal received a single IP injection of STZ freshly dissolved in ice-cold sterile saline 0.9% at a dose of 100 mg/kg [21] to induce type 1 diabetes mellitus.

### 2.3. Blood Glucose Quantification

Three days after diabetes induction, the level of fasting blood glucose (FBG) was detected by glucometer (Bionime GM300, Swiss Design, Berneck, Switzerland). Rats with an FBG level above 200 mg/dL were considered diabetic and selected [22]. FBG was evaluated every week during the experiment.

### 2.4. Blood Sampling

Peripheral blood samples were collected from Groups I and IIa in EDTA-containing tubes and centrifuged at 2500× *g* for 15 min at 4 °C within 3 h from collection. Aliquots of plasma samples were frozen at −20 and at −80 °C for biochemical assessment and miRNA extraction, respectively.

### 2.5. Intravitreal Administration of FSK

The rats were randomly selected and anesthetized with an IP injection of ketamine (120 mg/kg) and xylazine (16 mg/kg). A 1% tropicamide saline solution (Mydrapid, Alexandria Co., Alexandria, Egypt) and 0.4% benoxinate hydrochloride (Benox, Eipico, Tenth of Ramadan City, Egypt) eye drops were topically administered to dilate and anesthetize the right eyes, respectively. FSK (F20685; RPI Corp, Mount Prospect, IL USA); 6 nmol/eye (corresponding to about 100 μM as the final concentration) [18], was slowly injected into the right eye vitreous cavity through the pars plana using a 33-gauge microsyringe (Hamilton, Reno, NV, USA) at a volume of a controlled injection volume of 2.5 µL per eye using a calibrated Hamilton microsyringe to ensure consistency across all animals. Contralateral left eyes served as controls. The rats were then placed on a heating pad during the procedure and recovery period. While recovering from anesthesia, the rats were placed in their home cages, and B.N.P. Triple Antibiotic (Bacitracin, Neomycin, Polymyxin B; Akorn Animal Health, Inc., Lake Forest, IL, USA) and artificial tear ophthalmic ointment were applied to the cornea to prevent corneal dryness and infection.

### 2.6. Total Antioxidant Capacity (TAC) and Total Peroxide (TP) Assessment

TAC was assessed colorimetrically in the plasma via the commercially available kit (Bio-Diagnostics, Cairo, Egypt). Detection of TP was performed colorimetrically through an enzymatic reaction that includes the oxidation of ferric–xylenol orange into a colored product. The ability of antioxidants in the plasma to neutralize reactive oxygen species led to the creation of a quantifiable colored product proportionate to the sample’s total antioxidant defense capacity (TAC), which was determined using a colorimetric assay [23].

### 2.7. Lipid Profile Estimation

Triglycerides (TGs), total cholesterol, and high-density lipoprotein cholesterol (HDL-C) were detected by commercially available kits (Bio-Diagnostics, Cairo, Egypt). Low-density lipoprotein cholesterol (LDL-C) levels were calculated [24].

### 2.8. MiRNA Extraction and Reverse Transcriptase

MiRNA was extracted from the plasma of each rat using the miRNeasy Mini Kit (Qiagen, Hilden, Germany; Cat. No. 217004). The purity and concentration of RNA were determined by biotech nanodrop. Poly A polymerase enzyme (New England Biolabs, lpswich, MA, USA; Cat. No. M0276L) was used to add a poly A tail to small non-coding miRNA. Reverse transcription was conducted with the Thermo Scientific Revert Aid Reverse kit (Thermo Fisher Scientific, Waltham, MA, USA), and cDNA was collected after the transcription.

### 2.9. Quantitative Real-Time PCR (qRT-PCR) Analysis for miRNA-200b

qRT-PCR was performed on the 7500 fast real-time PCR system (Applied Biosystems, Carlsbad, CA, USA) using the SensiFAST SYBR® Lo-ROX Kit (Bioline, Memphis, TN, USA; Cat. No. BIO-94020). An initial denaturation for 2 min at 95 °C was followed by 40 cycles of denaturation for 10 s at 95 °C, with an annealing/extension at 60 °C for 30 s. The relative transcript levels of miRNA-200b were calculated using the equation of fold change = 2^−∆∆ct^. U6-snRNA acted as an internal control, and all primers were synthesized by Invitrogen (Carlsbad, CA, USA) [25,26]. Sequences of the primers are illustrated in Table 1.

### 2.10. Immunohistochemistry

Seven days after the FSK injection, Group IIb rats were euthanized and intracardially perfused first with phosphate-buffered saline (PBS) and then 4% paraformaldehyde (PFA) in PBS. Both treated and contralateral control eyes of each animal were gently enucleated. For sections, eyes were post-fixed overnight and cryoprotected with 30% sucrose at 4 °C for 24–48 h. Cryosections at 20 ųm thickness were placed on slides and allowed to dry at Room Temperature (RT) for 10 min.

Air-dried sections were washed 3 times in PBS, and then blocked with blocking solution (3% BSA and 0.2% Triton X-100 in PBS) for 1 h at RT. Primary antibodies were applied to the sections for at least 24 h at 4 °C, followed by three rinses with PBST buffer (0.2% Triton X-100 in PBS). The following primary antibodies were used: NeuN (GTX30773; mouse monoclonal; GeneTex; Friesing, Germany, 1:100) to label neurons, Cleaved Caspase-3 (Asp175) (#9661; rabbit monoclonal; Cell Signaling Technology, Danvers, MA, USA; 1:400) to label apoptotic cells, IBA1 (GTX632426; mouse monoclonal; GeneTex, Friesing, Germany; 1:200) to label microglia, and GFAP (MA5-12023; mouse monoclonal; Thermo Fisher Scientific, Waltham, MA, USA; 1:200) to label astrocytes.

Tissues were subsequently incubated with Alexa Fluor 555- or 488-conjugated secondary antibodies (Thermo Fisher Scientific, Waltham, MA, USA) for 3 h at RT. Nuclei were counterstained with Hoechst 33342 (H3570; Themo Fisher). After triple washing with PBST buffer, the samples were mounted and cover-slipped with 2.5% PVA-DAPCO anti-fading medium and examined by an Olympus U-RFL-T immunofluorescent microscope.

### 2.11. Cell Counting

NEUN+ cells were quantified from 6 micrograph images (three continuous images from each side of the optic nerve head) as previously described [7]. A Cell Counter software plugin in the ImageJ program (version 1.54) (Leica Q 500 MCO; Wetzlar, Germany) was used for cell counting. On each retinal section, marker-positive cell bodies were evaluated. The total number of marker-positive cells for each retina is the average of all analyzed sections obtained at comparable locations from both the superior and inferior retina.

### 2.12. Statistical Analysis

Data was analyzed using the GraphPad Prism software version 8 (GraphPad Inc., La Jolla, CA, USA). Differences between groups were compared using one-way ANOVA followed by Tukey’s test. Pearson correlation analysis was also performed to explore potential relationships between miR-200b and biochemical parameters. The data were presented as mean ± SD. Differences were statistically significant at *p* value ≤ 0.05. An a priori power analysis for the primary outcome, based on a one-way ANOVA with three groups, α = 0.05, and 80% power, indicated that a total sample size of 18 rats would be sufficient to detect a large effect size (Cohen’s f = 0.8).

## 3. Results

### 3.1. STZ-Induced Diabetes in Rats

Eight weeks after diabetes induction, the fasting blood glucose (FBG) levels were significantly higher in diabetic rats (DM and DM + FSK) compared to the control rats (*p* < 0.0001) (Table 2). In addition, the diabetic rats showed polyphagia, polydipsia, and polyuria, which are considered the typical manifestations of DM, suggesting the induction of DM in rats.

### 3.2. Diabetes Induced a State of Oxidative Stress and Altered Lipid Profile

Diabetic rats revealed significantly decreased levels of TAC (*p* < 0.0001) and increased levels of TP (*p* < 0.0001) compared to the control group. DM + FSK rats displayed a slight improvement in TAC and TP compared to untreated DM rats, but differences were not statistically significant (Table 2).

Lipid profile analysis revealed significantly increased levels of TGs (*p* < 0.0001), total cholesterol (*p* < 0.0001), and LDL-C (*p* < 0.0001), which were associated with significantly reduced levels of HDL-C (*p* < 0.0001) in the diabetic group when compared with the control rats. DM + FSK rats showed no significant changes in TGs, total cholesterol, LDL-C, or HDL-C compared to DM rats (Table 3).

### 3.3. Diabetes Increased the Levels of miR-200b

**Figure 1:**
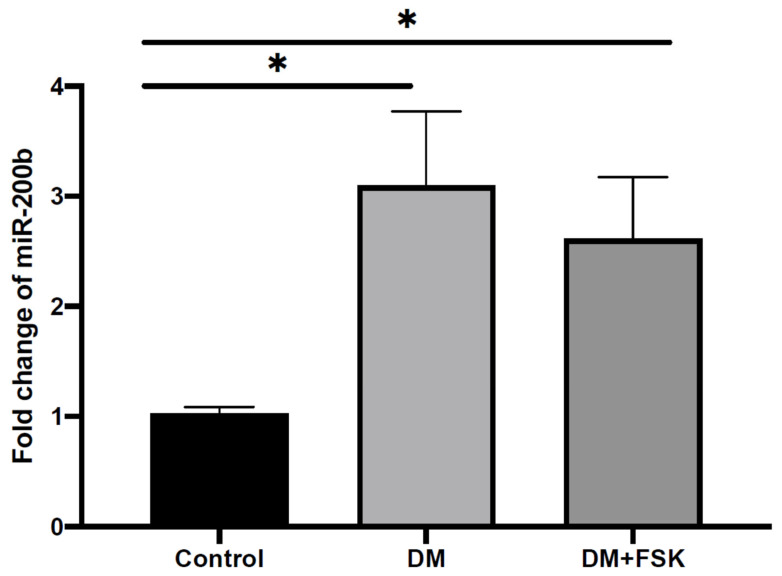
Fold change in miR-200b in the studied groups. Each value represents the mean ± SD. * *p* < 0.05 vs. the control group.

The diabetic rats showed a significantly higher plasma expression of miR-200b than the control rats (*p* < 0.0001). FSK treatment slightly lowered miR-200b, but the change was not statistically significant (Figure 1).

Interestingly, a significant positive correlation was found between the expression of miR-200b and the levels of blood glucose (r = 0.833, *p* < 0.001), TP (r = 0.754, *p* < 0.001), TGs (r = 0.871, *p* < 0.001), total cholesterol (r = 0.865, *p* = 0.001), and LDL-C (r = 0.837, *p* = 0.001). Conversely, a significant negative correlation was found between the expression of miR-200b and the levels of TAC (r = −0.847, *p* < 0.001) and HDL-C (r = −0.802, *p* < 0.001). Correlation analyses were performed for exploratory purposes and should be interpreted with caution due to the limited sample size.

### 3.4. FSK Alleviates Neuron Death in Diabetic Retinopathy (DR)

We next examined whether DR-induced neuron death can be mitigated by FSK. The rats were randomly divided into two groups (n = 6). One group received intravitreal FSK (6 nmol/eye) in the right eye, while the contralateral left eye served as an internal control. The second group received IP saline (0.5 mL) as a vehicle control.

**Figure 2:**
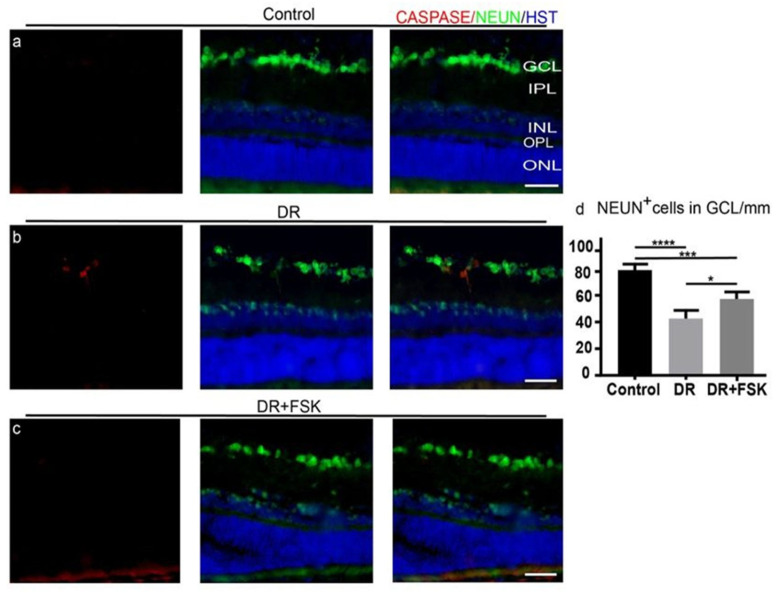
FSK mitigates neuron death in DR. (**a**) Caspase 3 negative staining of ganglion cells (NEUN^+^) in the control section. (**b**) In DR, NEUN^+^ neurons are co-labeled with Caspase 3, unlike FSK-treated neurons (**c**). Scale bars, 50 μm. (**d**) Quantification of NEUN^+^ cells. Data are presented as mean ± SD per mm retinal length. Statistical analysis was performed by one-way ANOVA with post hoc Tukey’s test. **** *p* < 0.0001; *** *p* = 0.0008; and * *p* = 0.01.

One week after FSK injection, immunohistochemistry was performed to detect NEUN+ and Cleaved Caspase 3 + neurons, which were analyzed by fluorescent microscopy (Figure 2a–c). Furthermore, NEUN+ cells in the ganglion cell layer (GCL) were quantified to determine the effect of FSK on neuron survival (Figure 2d).

In DR, a fraction of NEUN+ neurons in the GCL were co-labeled with Cleaved Caspase 3, a marker for apoptosis, unlike the control and FSK-treated neurons (Figure 2a–c). Quantification showed that DR caused a significant decrease in neuron cell number when compared to the control group (*p* < 0.0001). Moreover, intravitreal FSK treatment significantly improved neuronal survival in DR (*p* = 0.01) (Figure 2d).

**Figure 3:**
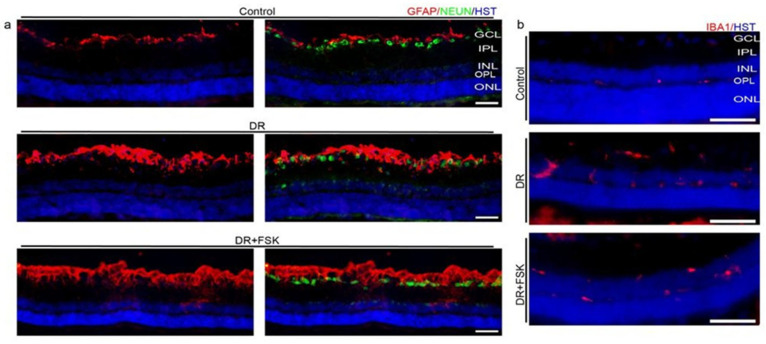
Effect of FSK on glial activation. (**a**) When compared to the controls, GFAP expression increases in both DR- and FSK-treated eyes. (**b**) DR induces robust activation of IBA1+ microglia cells, and FSK treatment fails to change their morphology and retinal layer distribution.

We further examined glial cell morphology in DR by immunostaining microglia and astrocytes with Ionized calcium-binding adaptor molecule 1 (IBA1) and glial fibrillary acidic protein (GFAP), respectively. GFAP expression was seen to increase in DR, when compared to the controls (Figure 3a). Likewise, microglia/macrophage morphology and distribution among retinal layers noticeably changed in DR as IBA1+ cells showed soma enlargement with thickening of their processes (Figure 3b). FSK did not produce observable qualitative changes in GFAP expression or IBA1+ cell morphology under the conditions tested (Figure 3a,b).

## 4. Discussion

According to the WHO, 34.6% of diabetics worldwide are complicated with DR [27,28], while the incidence in Egypt is 42% among Egyptian diabetics [29,30].

In this study, STZ at a dose of 100 mg/kg succeeded in the induction of diabetes in rats, as evidenced by the highly significant hyperglycemia developed in those rats versus the controls. After 8 weeks, hyperglycemia was complicated by an enhanced oxidative stress state in the form of significantly decreased levels of TAC and significantly elevated levels of TP in the plasma. In addition, diabetic rats also showed dyslipidemia manifested with elevated TGs, total cholesterol, and LDL-C, and reduced HDL-C. Sustained hyperglycemia could lead to ischemia in the retina that stimulates angiogenesis and neovascularization. The leaky vessels result in hemorrhage and detachment of the retina that may cause blindness [31].

Diabetes-induced abnormal lipid metabolism results in increased risk for the development of DR [32]. TGs and TG-rich lipoproteins were considered causal factors for microvascular complications of diabetes [33]. Although the exact mechanism is not understood, persistent hyperglycemia and hyperlipidemia in diabetes cause vascular endothelium damage with endothelial dysfunction, which leads to microvascular retinal complications [34]. Destruction of the blood–retinal barrier, which might occur in diabetes, enhances the entry of lipoprotein particles into the retina with consequent increased levels of retinal cholesterol, which is involved in the pathogenesis of DR [32]. In contrast, Takele et al. [35] did not observe obvious variations in the levels of TGs, TC, and HDL-C in patients with and without DR. They only found a slightly increased LDL-C in patients with DR.

A major role of oxidative stress was reported in the pathogenesis of DR. The retina is highly susceptible to oxidative stress due to the increased demands of energy and light exposure [36]. Increased reactive oxygen species (ROS) in diabetic retina impairs cellular signaling, leading to damage of retinal cells and ending in DR [37]. Under hyperglycemic conditions, the increased formation of advanced glycation end products in the mitochondria impairs the cells in the vasculature, resulting in microvascular abnormalities. This lifelong impairment is responsible for the lack of maintenance of vascular homeostasis by the endothelium [38]. Furthermore, hyperglycemia induces epigenetic modifications that suppress the antioxidant defense system, with an imbalance between the formation and scavenging of ROS. Increased accumulation of ROS results in mitochondrial damage, apoptosis, lipid peroxidation, and structural and functional changes in the retina [39].

The current study also illustrated that the diabetic rats showed an upregulated plasma expression of miR-200b when compared to the control rats. Positive correlations were also found between miR-200b levels and levels of glucose, TP, TGs, total cholesterol, and LDL-C, while negative correlations were found between miR-200b levels and levels of TAC and HDL-C. These findings suggest a potential association between miR-200b and metabolic and oxidative stress parameters; however, these correlations are exploratory and warrant confirmation in larger studies. The expression of miR-200b remains controversial under high glucose circumstances. In support of our results, microvascular endothelial cells subjected to hyperglycemia showed a consistent rise in the expression of miR-200b. This epigenetic dysregulation of miR-200b secondary to hyperglycemia is a major cause of endothelial dysfunction and diabetic vasculopathy, and hence DR [40]. Injury downregulates miR-200b, which is necessary to stimulate wound angiogenesis [41]. However, a hyperglycemic state is associated with loss of this injury-induced suppression of miR-200b [12]. Murray and colleagues reported that miR-200b expression is enhanced in spontaneous diabetic mice, followed by downregulation of the Oxr1 gene, which stimulated oxidative stress in Müller cells [42]. In contrast, miR-200b expression was found to be decreased in the retina of diabetic mice and endothelial cells exposed to high glucose conditions [43]. MiR-200b has also been reported to attenuate the development of diabetic retinopathy (DR) by decreasing the expression of its target gene, VEGFA. In diabetes, reduced miR-200b levels in the retina are associated with microvascular retinal leakage and blood–retinal barrier (BRB) disruption [44]. The discrepancy between the increased circulating miR-200b observed in the present study and the reduced retinal miR-200b expression reported by others likely reflects tissue-specific regulation and disease stage-dependent effects. Circulating miRNAs may be released from injured retinal or vascular cells under hyperglycemic and oxidative stress conditions and therefore do not necessarily parallel tissue expression levels. Accordingly, elevated plasma miR-200b may have potential as a biomarker of diabetic retinal stress, while reduced retinal miR-200b may contribute to local loss of protective signaling [12].

The current research assessed the potential neuroprotective ability of intravitreal FSK on DR. We found that the retina of diabetic rats has a significant reduction in neuron cell number when assessed by immunostaining with Caspase 3. Apoptosis of retinal cells is one of the important pathological responses at the early stage of DR. Moreover, apoptosis is a leading mechanism of retinal neurodegeneration and nerve tissue injury [45]. High glucose exposure of the cells in the retina results in aggravation of oxidative stress, leading to an increase in Caspase 3 activity, which accelerates cellular death [39]. This cellular loss in RGCs is involved in DR pathogenesis [46]. In contrast, intravitreal FSK improved neuronal survival as Caspase 3 expression was significantly reduced. This could be based on the known antioxidant ability of FSK [47]. Consistent with our results, FSK decreased the mRNA expression of Caspase 3 and inhibited the activation of apoptosis, and also reduced the formation of ROS, and hence protected against hair cell loss induced by cisplatin [48]. FSK acts via increasing levels of intracellular cAMP, which is responsible for the regulation of various cellular processes, such as cellular death and survival [49]. In a previous study, cAMP inhibited arsenic trioxide-induced activation of Caspase 3 and apoptosis of acute promyelocytic leukemia cells [50].

The results also indicated that diabetic retinae had altered glia cells and changed the microglia/macrophage morphology and distribution among retinal layers. Hyperglycemia activates microglia and astrocytes, which is correlated with the elevated oxidative stress and inflammatory markers [51]. Activation of glial cells may contribute to neurodegeneration via increased oxidative stress and mitochondrial fragmentation in RGCs [52]. Dysregulation of metabolic pathways induced by diabetes results in elevated ROS that causes astrocyte activation [53], which is consistent with the increased expression of GFAP observed in our study. FSK did not produce observable qualitative changes in GFAP expression or IBA1+ cell morphology under the conditions tested.

Since FSK was administered locally, systemic changes in circulating miRNAs are likely limited. In our study, intravitreal FSK did not significantly alter circulating miR-200b; however, several biologically plausible mechanisms could modulate miRNA regulation within the retina. FSK activates adenylate cyclase, raising intracellular cAMP levels, which can influence transcription factors such as CREB and engage PKA-dependent signaling pathways that support neuronal survival and cellular homeostasis [54]. Additionally, FSK has reported antioxidant and anti-inflammatory effects, reducing reactive oxygen species and improving redox balance, which may indirectly help normalize miR-200b expression under diabetic oxidative stress [55]. These mechanisms remain theoretical and require direct experimental verification. Future studies should investigate whether FSK affects miR-200b expression and clarify how oxidative stress, cAMP signaling, and microRNA regulation interact in diabetic retinopathy [55].

### Limitations

There are a number of limitations to consider when interpreting the results of this study. Retinal tissue was not evaluated for miR-200b expression, so the local effects of FSK on retinal miRNA remain unknown. While intravitreal FSK improved neuronal survival in the retina, it did not appear to significantly alter systemic circulating miR-200b or other metabolic markers, suggesting its effects may be largely local. Glial activity was assessed qualitatively through immunofluorescence rather than quantitatively, which may limit the precision of these observations. U6-snRNA was used as the internal control for circulating miRNAs; however, its stability across experimental groups was not experimentally validated. Finally, the relatively small sample size may reduce statistical power and the generalizability of the findings. The relatively small sample size (n = 6 per group) may increase the risk of Type II error, potentially obscuring subtle effects, particularly in metabolic and glial outcomes. Therefore, non-significant findings should be interpreted with caution. Future studies should include direct assessment of retinal miRNAs, quantitative evaluation of glial and oxidative responses, validation of reference gene stability, and larger cohorts to confirm these results and clarify underlying mechanisms.

## 5. Conclusions

In conclusion, our findings demonstrate that hyperglycemia, oxidative stress, hyperlipidemia, and elevated circulating miR-200b are associated with the development of DR in the STZ-induced diabetic rat model. Intravitreal FSK provided neuroprotection by improving retinal neuronal survival and reducing Caspase 3 expression, indicating inhibition of apoptosis. While FSK slightly lowered circulating miR-200b, this change was not statistically significant, suggesting that its effects may be local within the retina. Future studies should directly assess retinal miR-200b expression and investigate whether modulation of this miRNA contributes to the neuroprotective actions of FSK in diabetic retinopathy.

## Figures and Tables

**Table 1:** Primer names and sequences.

Primer Sequence	Primer Name
5′-CGCTTCGGCAGCACATATAC-3′	Forward U6
5′-TTCACGAATTTGCGTGTCAT-3′	Reverse U6
5′-CTCCCTAAAGCCTCCCACC-3′	Forward miRNA-200b
5′-AGGGCTTTCTGCTGTTGTCC-3′	Reverse miRNA-200b

**Table 2:** Levels of blood glucose, plasma total antioxidant capacity (TAC), and plasma total peroxide (TP) in the studied groups.

	Control	DM	DM + FSK
Blood glucose (mg/dL)	74.17 ± 7.36	348.30 ± 32.95 *	353.75 ± 33.64 *
Plasma TAC (µmol/mL)	1.10 ± 0.13	0.47 ± 0.05 *	0.56 ± 0.09 *
Plasma TP (µmol/mL)	0.10 ± 0.02	0.19 ± 0.01 *	0.17 ± 0.02 *

Each value represents the mean ± SD. * *p* < 0.05 vs. the control group.

**Table 3:** Levels of triglycerides (TGs), total cholesterol, high-density lipoprotein cholesterol (HDL-C), and low-density lipoprotein cholesterol (LDL-C) in the studied groups.

	Control	DM	DM + FSK
TGs (mg/dL)	80.22 ± 7.88	135.70 ± 13.86 *	133.6 ± 6.83 *
Total cholesterol (mg/dL)	81.17 ± 6.97	181.30 ± 14.29 *	176.52 ± 11.8 *
HDL-C (mg/dL)	40.99 ± 4.27	22.43 ± 1.48 *	24.28 ± 3.09 *
LDL-C (mg/dL)	24.14 ± 5.69	131.80 ± 16.96 *	133.6 ± 13.7 *

Each value represents the mean ± SD. * *p* < 0.05 vs. the control group.

## Data Availability

All data are available upon request.
